# Phytochemical Composition and Skin-Friendly Activities of the Ethyl Acetate Fraction in *Ophioglossum vulgatum* Linn., an In Vitro Study

**DOI:** 10.3390/ph18030345

**Published:** 2025-02-27

**Authors:** Sihan Feng, Zhiguang Huang, Yichen Cao, Zixuan Huang, Chen Xu, Yibo Zeng, Yuhang Xu, Lijian Zhu, Bin Ding

**Affiliations:** 1 School of Life Sciences, Zhejiang Chinese Medical University, Hangzhou 310053, China; fsihan0203@zcmu.edu.cn (S.F.); huangzg0607@163.com (Z.H.);; 2 The Second Clinical Medical College, Zhejiang Chinese Medical University, Hangzhou 310053, China; 3 Shanghai Institute of Biochemistry and Cell Biology, Center for Excellence in Molecular Cell Science, Chinese Academy of Sciences, University of Chinese Academy of Sciences, Shanghai 200031, China; zhulijian2024@sibcb.ac.cn

**Keywords:** *Ophioglossum vulgatum* Linn., ingredients, bioactivity, antioxidative, anti-inflammation, antibacteria

## Abstract

**Background**: *Ophioglossum vulgatum* Linn. is a medical herb widely distributed in Southwest China. It has been used for the treatment of various diseases, including wounds or dermatitis, since ancient times, but little is known about its pharmacological and pharmaceutical chemistry. **Methods**: The ethyl acetate fraction of *O. vulgatum* (OpvE) was prepared with the reflex extraction and fractional extraction method. Its components were detected and identified with the UPLC-Q/TOF-MS system and the SCIEX OS database. The related biological activities and the underlying mechanisms were predicted by computational analysis. HaCaT cells were treated with gradient concentrations of OpvE, and a CCK-8 assay was performed to determine the cell viability. The OpvE-pretreated HaCaT cells were exposed to H_2_O_2_ or LPS for antioxidative and anti-inflammatory assessment. DPPH, GSH, SOD, and MDA kits were used to evaluate oxidative stress. A serially diluted microbiota assay and a disc diffusion assay were used to evaluate anti-*Staphylococcus aureus* activities. The transcription of genes was semi-quantitatively studied by reversed real-time PCR. Protein levels were determined with western blotting. **Results**: The extract ratio of OpvE was 2.00 ± 0.12% (g/g). A total of 21 ingredients were identified. The computational analysis found that the PI3K/Akt signaling pathway might be a crucial target of OpvE. OpvE (7.5~125 μg/mL) stimulated HaCaT cell proliferation and migration by stimulating the over-expressed collagen type I alpha 1 Chain (*COL1A1*) and fibronectin 1 (*FN1*) and upregulating PI3K/AKT/GSK3-β signaling pathway. In the antioxidative assay test, 250 μg/mL OpvE scavenged obvious 97.28% DPPH-released free radicals. Pretreatment of OpvE inhibited H_2_O_2_-induced oxidative stress and protected against LPS-induced inflammatory injury by respectively regulating the Nrf2/HO-1/COX2 and TLR4/MYD88 signaling pathways. OpvE also showed anti-*S. aureus* properties with a MIC of 1.2 mg/mL, and with this concentration, OpvE produced an 8.3 ± 0.16 mm inhibition zone on a bacterial plate. **Conclusions**: This work highlighted the phytochemical character and some bioactivities, as well as the underline mechanism, which would support the further studies and application of *O. vulgatum* Linn.

## 1. Introduction

*Ophioglossum vulgatum* Linn. (*O. vulgatum*), a vegetation genus including some inconspicuous ferns, belongs to Polypodiopsida [1]. Its name combines the Greek words ophis (snake) and glossa (tongue), referring to the shape of the spore spike of this genus. Also, it was considered by ancient people to resemble an arrow due to its shape. *O. vulgatum* is a popular folk medicine and nutrient supplement in Southwest China [2]. Conventionally, *O. vulgatum* had many uses in traditional Chinese medicine, such as curing carbuncle, furuncle, and scabies or eliminating the symptoms of injuries from falls, chest and abdomen pain, snake or dog bites, and stomach pain [3]. In recent decades, few scientific studies have been conducted to elucidate the pharmacological activities of *O. vulgatum*.

*Ophioglossum* has previously been reported to detoxify, promote blood circulation, and ameliorate fatigue [4,5]. In addition, recent studies have proposed *O. vulgatum* in investigations for dermatological [6], hemostatic [7], antiparasitic [8], antivirus [9], and wound healing [10,11] purposes. Mao Lingfei and her colleagues illustrated that an ethanol extract of *O. vulgatum* can significantly promote the expression of epidermal growth factor in the marginal tissue of acetic acid-induced gastric ulcers in rats [12]. Notably, Xu et al. reported that a Pickering emulsion of *O. vulgatum* extract showed a potential application as a cosmetic product [13]. Our previous work indicated the hair growth-improving capability of the ethyl acetate fraction in *O. vulgatum* (OpvE), which suggested the skin-friendly activities of OpvE [14]. However, the phytochemical character and biological activities are still unclear. Hence, in this study, we identified the chemical constituents of OpvE with a UPLC-Q/TOF-MS analysis. Skincare and anti-aging prominently involve processes of antioxidation and anti-inflammation, among others [15]. Hence, we verified the skin-friendly activities and the underlying mechanism of OpvE with the HaCaT cell assay tests in vitro.

## 2. Results

### 2.1. OpvE Preparation and Ingredients Identification

**Figure 1:**
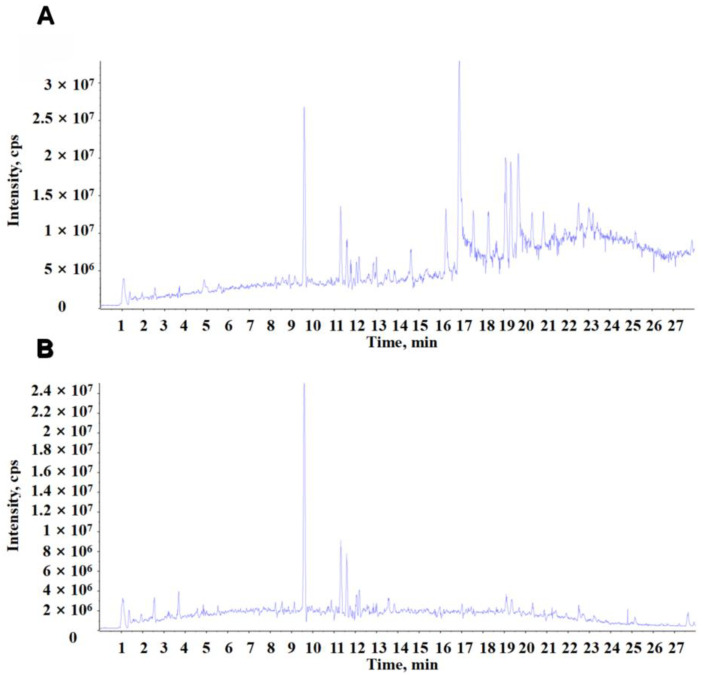
Total ion chromatograms (TICs) of OpvE. (**A**) TIC of OpvE in positive ion mode. (**B**) TIC of OpvE in negative ion mode.

A total of 300 ± 19 mg OpvE was extracted from 15 g of *O. vulgatum* powder. The extractive efficiency was 2.00 ± 0.12% (g/g). The chemical components in OpvE were identified with a UPLC-Q/TOF-MS/MS system. The signals of typical total ion chromatograms (TICs) of nonvolatile ingredients in OpvE are presented in positive and negative ion modes in Figure 1. A total of 17 compounds in the positive (Table A1) and 5 in the negative ion mode (Table A2), were identified by comparing and filtering with the standard primary accurate mass, the isotope distribution ratio, and MS/MS of the compound TCM MS/MS Library in the TCM secondary database of the SCIEX OS. Luteolin was found in both positive and negative ion modes. Betaine, luteoloside, liquiritin, and 6-gingerol were only found in the negative ion mode.

### 2.2. Bioinformatic Analysis and Pharmacological Activity Prediction of OpvE

**Figure 2:**
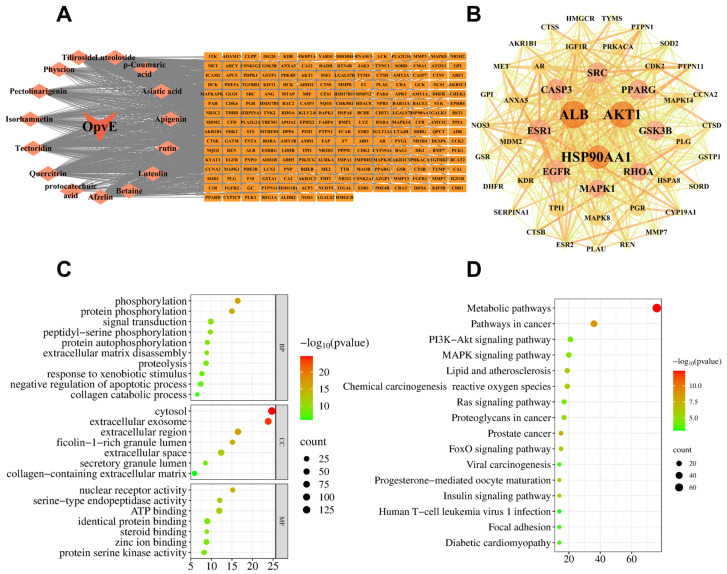
Computational analysis of OpvE. (**A**) The ingredients–targets network of OpvE with 218 predicted targets. (**B**) PPI networks of 47 highly focused candidate genes of OpvE. The size and color indicated the significance. (**C**) The top 10 biological functions and top 7 cell components and molecular functions of the GO enrichment analysis. (**D**) The top 16 signaling pathways of KEGG enrichment analyses. The horizontal axis represents the proportion of enriched items. The proportion of enriched items is depicted on the horizontal axis, while each item is shown on the vertical axis. The size of the dots and color corresponds to the number of enrichment targets and *p*-values.

Some internet-based pharmacological tools and databases were used to predict the activity of OpvE. Firstly, the target genes were screened and selected in the DrugBank database and the UniProt database. A total of 218 targets of OpvE were obtained and shown with an ingredients-target network (Figure 2A). After analyzing with a sequence-based and topology-based method for candidate gene selection as previously described [16], 47 candidate genes were ultimately selected (Figure 2B).

We input these candidates into the DAVID database and selected 615 GO genes, of which 420 related to biological function (BP), 56 to cell component (CC), and 139 to molecular function (MF), and the top 10 were shown in Figure 2C. BP items were mainly related to phosphorylation, protein phosphorylation, signal transduction, and peptidyl-serine phosphorylation. CC items were highly focused on the cytosol, the extracellular exosome, the extracellular region, and the ficolin-1-rich granule lumen. The highlighted MFs included nuclear receptor activity, serine-type endopeptidase activity, ATP binding, and identical protein binding.

Simultaneously, the KEGG analysis was performed on the 218 targets, and 16 pathways were selected with *p* < 0.05 as the criterion (Figure 2D). The top three results were metabolic pathways, pathways in cancer, and PI3K/AKT signaling pathways. To our knowledge, PI3K/AKT regulates multiple biological processes, such as cell proliferation and migration. Hence, we tried to prove our findings with the following experiment.

### 2.3. Cell Proliferation-Promoting and Wound-Healing Capability of OpvE

**Figure 3:**
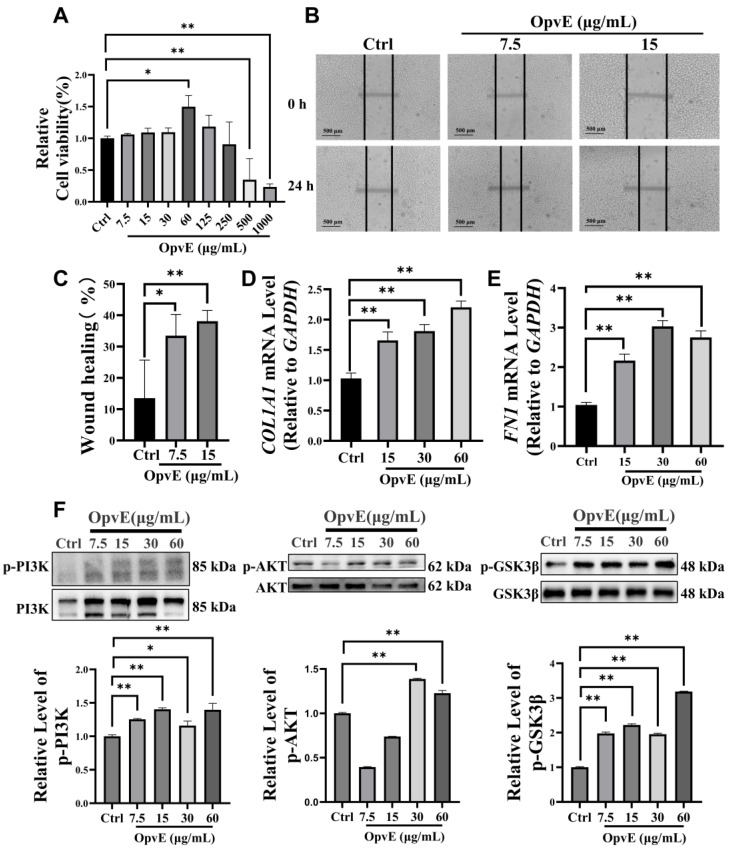
OpvE promoted cell proliferation and wound healing. (**A**) The gradually increased OpvE affected the proliferation of HaCaT cells. The transcription of *COL1A1* (**B**) and *FN1* (**C**) in HaCaT cells assays with 15, 30, and 60 μg/mL OpvE. The image (**D**) and the semi-quantitative analysis (**E**) of wound healing assay tests. Western blotting (**F**) and the semi-quantitative analysis of p-AKT/AKT, p-PI3K/PI3K, and p-GSK3β/GSK3β levels in 7.5, 15, 30, 60 μg/mL OpvE-treated HaCaT cell assays. All the values are denoted as means ± SD (*n* = 3). * *p* < 0.05, ** *p* < 0.01 vs. the Ctrl.

To investigate the cell-promoting capability of OpvE, we evaluated the viability of OpvE-treated HaCaT cells with the CCK-8 method. Cells were treated with 7.5 to 1000 μg/mL OpvE in this study. As the concentration increased from 7.5 to 60 μg/mL, we observed the improving effects of OpvE on cell viability. Only 60 μg/mL OpvE produced a significant effect. This effect decreased as the concentration continually increased to 250 μg/mL. In comparison with that of the Ctrl assay, a high concentration of OpvE (≥500 μg/mL) showed significant cell toxicity activities in a dose-dependent manner (Figure 3A).

Then, we performed a wound-healing assay to evaluate the effects of 7.5 and 15 μg/mL OpvE on the migration of HaCaT cells. Treatment with OpvE significantly increased cell migration (Figure 3B,C). In 24  h, the wound healing ratio of HaCaT assays, with the presence of 7.5 μg/mL and 15 μg/mL OpvE, was increased by 19.9% and 24.5%, respectively, in a concentration-dependent manner. The transcription of collagen type I alpha 1 chain (*COL1A1*) and fibronectin 1 (*FN1*), two hallmarks of cell proliferation and migration, were stimulated by OpvE (15~60 μg/mL) treatment in HaCaT cells (Figure 3D,E). This proved that OpvE can significantly enhance cell proliferation and migration.

To confirm the predicted results, we evaluated the levels of p-AKT, p-PI3K, and p-GSK3-β in OpvE-treated HaCaT cells. As shown in Figure 3F, OpvE (7.5~60 μg/mL) stimulated the PI3K/AKT/GSK3-β signaling pathway. Multiple evidence indicates that a harmonious redox and inflammatory status is critical for skin health. Therefore, we employed H_2_O_2_- and LPS-treated HaCaT cell assays to evaluate the protective effects of OpvE.

### 2.4. OpvE Protects the H_2_O_2_-Induced Cell Damage

**Figure 4:**
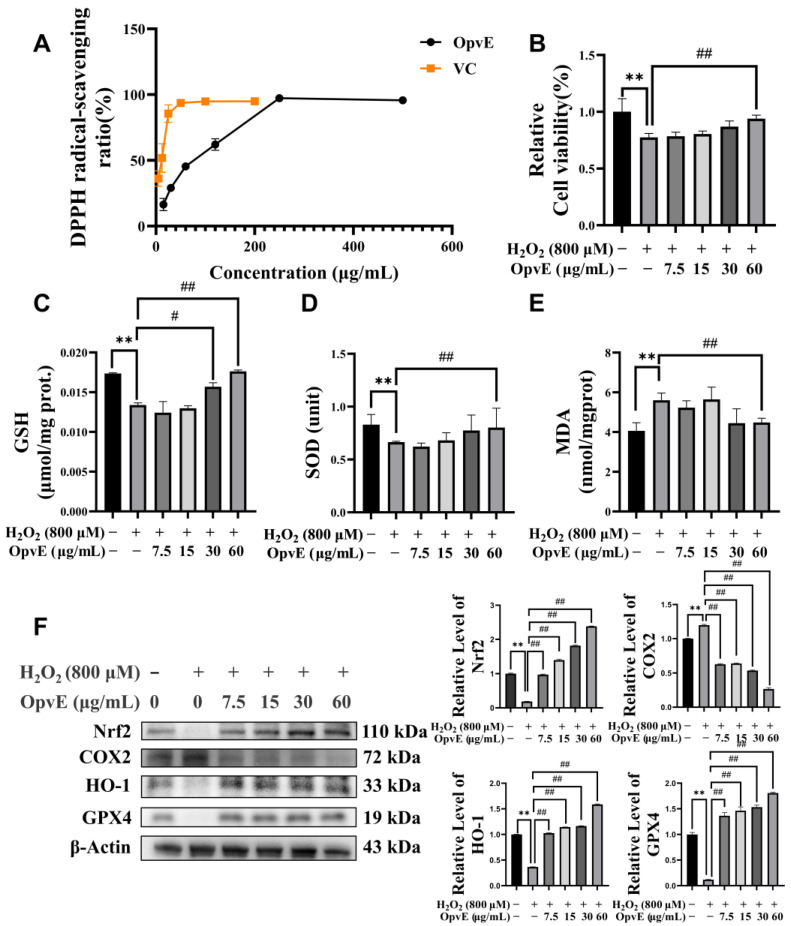
The antioxidative capability of OpvE. (**A**) The free radical-scavenging activity of OpvE and vitamin C was comparatively evaluated with DPPH assay tests. (**B**) The pretreatment of OpvE was able to protect the H_2_O_2_-induced reduced cell viability by improving the GSH (**C**) and SOD (**D**) levels and downregulating the MDA levels (**E**) in HaCaT cells. The western blotting result and the semi-quantitative analysis (**F**) of the expression of Nrf2, COX2, HO-1, and GPX4 in the aforementioned various HaCaT cell assays. All the values are denoted as means ± SD (*n* = 3). ** *p* < 0.01 vs. UT group, # *p* < 0.05, ## *p* < 0.01 vs. H_2_O_2_ group.

A DPPH assay was applied to evaluate the antioxidative activities of OpvE, and the result is shown in Figure 4A. OpvE dose-dependently scavenged the DPPH-released free radicals. Based on this result, we further employed an H_2_O_2_-induced oxidative stress model to investigate the protective capability of OpvE. The result indicated that pretreatment of OpvE was able to protect H_2_O_2_-treated HaCaT cells by improving the abnormal levels of glutathione (GSH), superoxide dismutase (SOD), and malondialdehyde (MDA) (Figure 4B–E). Additionally, the expression of Nrf2, HO-1, and GPX4 in HaCaT cells was significantly restored by OpvE pretreatment, compared with the H_2_O_2_ group, and simultaneously, the expression of COX2 significantly decreased (Figure 4F). These results suggest that OpvE can ameliorate H_2_O_2_-induced oxidative stress by activating antioxidative factors such as GSH, SOD, and GPX4. Additionally, the Nrf2/HO-1/COX2 signaling axis was regulated.

### 2.5. OpvE Protects the LPS-Induced Cell Inflammatory Responds

**Figure 5:**
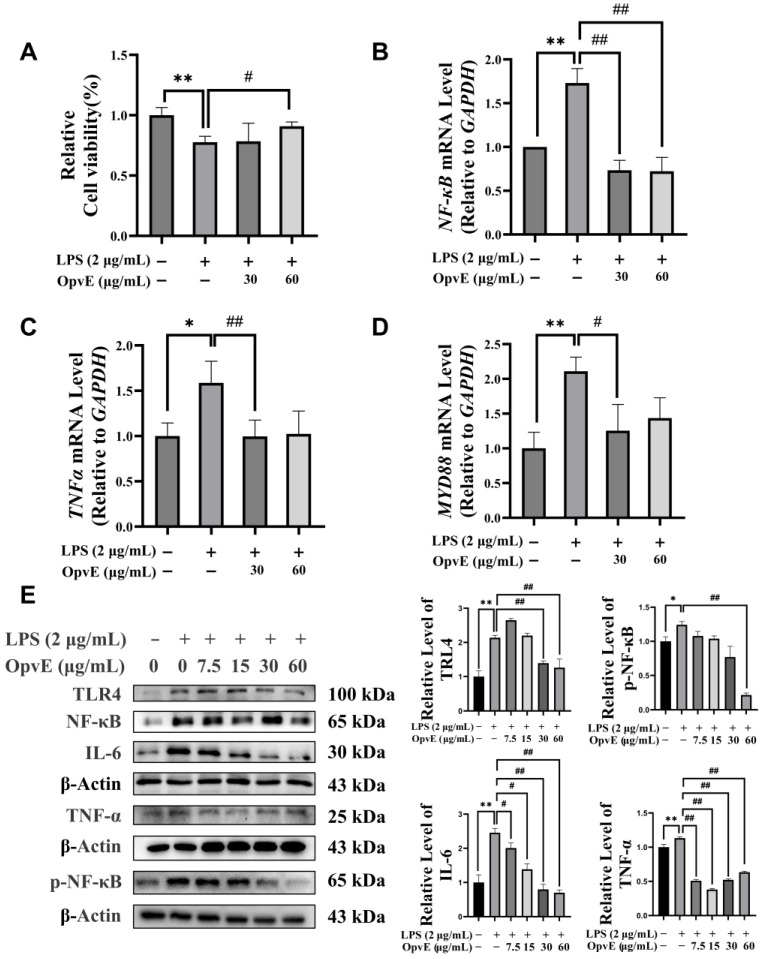
Anti-inflammatory activity of OpvE. (**A**) The pretreatment of OpvE was able to protect the LPS-induced reduced cell viability by attenuating the transcription of NF-κB (**B**), TNF-α (**C**), MYD88 (**D**), and the translation of TLR4, NF-κB, p-NF-κB, IL-6, and TNF-α (**E**). All the values are denoted as means ± SD (*n* = 3). * *p* < 0.05, ** *p* < 0.01 vs. UT group, # *p* < 0.05, ## *p* < 0.01 vs. LPS group.

Afterward, we analyzed the protective activity of OpvE on LPS-induced cell inflammatory injury. Pretreatment with 60 μg/mL OpvE significantly protected HaCaT cells (Figure 5A) by decreasing LPS-triggered high levels of inflammatory factors, such as NF-κB, TNF-α, and IL-6 (Figure 5B,C,E). The result of the real-time PCR and western blotting indicated that the expression of MYD88, TLR4, and p-NF-κB were regulated by OpvE (Figure 5D,E).

### 2.6. OpvE Inhibits the Growth of Staphylococcus aureus

*Staphylococcus aureus* is an opportunistic pathogen affecting skin health, and one of our interests was to test if OpvE has bacteriostatic activity. Hence, we determined the anti-*S. aureus* activity of OpvE with MIC value and disk diffusion assays. The OD_600_ of *S. aureus* broths with 0.3, 0.6, and 1.2 mg/mL OpvE were 0.261 ± 0.025, 0.184 ± 0.0132, and 0.082 ± 0.003, respectively. We calculated that the MIC value was 1.2 mg/mL. Based on this result, we increased the concentration of OpvE for disc diffusion tests. The inhibitory zones of OpvE are shown in Table 1. Compared with the positive control, vancomycin, the OpvE showed a very weak bacteriostatic activity.

## 3. Discussion

*O. vulgatum* is a kind of fern that has been used as a food and dietary supplement in Yunnan, Guizhou, and Sichuan Provinces in China since ancient times [17]. However, there have been few studies on the chemical composition and biological activity of *O. vulgatum* in recent decades. Previously, we found that the acetic ether fraction in the ethanolic extract of *O. vulgatum* (OpvE) can promote the growth of hair [14]. However, the phytochemical characteristics and underlining mechanisms were only partially elucidated. Hence, we used reflux extraction and fractional extraction to prepare the OpvE. Ethyl acetate, as a hydrophobic extractant, showed a high quantity of flavonoids, phenols, phenolic acids, and glycoside ligands in the crude plant extract [18]. The components in OpvE were detected with UPLC-Q/TOF-MS. A total of 21 ingredients (Table A1 and Table A2) were identified according to the TCM database of the SCIEX OS, 13 of which are flavonoids. The other three were protocatechuic acid, p-Coumaric acid, and 6-gingerol, which are phenolic acids and phenols. The remaining constituents include glycosides (flavone glycosides and triterpenoid saponins), anthraquinones (physcion), pentacyclic triterpenes (siatic acid, pedunculoside, and liriope muscari saponins C), and quaternary amine bases (betaine). The literature reports some glycosides in the extract of *O. vulgatum*, such as quercetin-3-O-[(6-caffeoyl)-β-glucopyranosyl (1 → 3) α-rhamnopyranoside]-7-O-α-rhamnopyr anoside, kaempferol-3-O-[(6-caffeoyl)-β glucopyranosyl (1 → 3) α-rhamnopyranoside]-7-O-α-rhamnopyranoside, quercetin-3-O-methylether [10], and 1,2-di-O-linolenoyl-3-O-β-d-galactopyranosyl-glycero [11]. However, we only found quercitrin. The aglucon of these ingredients was determined in our experiment. We propose that this might be the result of different extracting parameters.

In our experiment, luteolin has been identified both in the positive and negative models. This is a skin-friendly ingredient because of its antioxidant and anti-inflammatory activities in UVB-induced photoaging [19], wound healing [20], and psoriasis [21]. Rutin, for instance, is particularly renowned for its ability to strengthen blood vessels and reduce oxidative stress, making it an attempted supplement for clinical amelioration in atherosclerosis and diabetes [22,23]. Moreover, as the main component of the extract, the synergistic effect of rutin and quercetin further enhances the therapeutic potential, which is reflected in the skin-protective activity [24], including protecting skin cells against oxidative stress [25] or inflammations [26] induced by endogenous or exogenous factors [27]. This evidence encouraged us to evaluate the activities of OpvE with HaCaT cell assays in vitro.

We explored the cell proliferation improving capability of 7.5, 15, 30, 60, and 125 μg/mL OpvE, and only 60 μg/mL OpvE showed a significant effect (Figure 3A). This raised the question of whether OpvE, at low concentration, has a beneficial effect on the cell. Hence, we applied 7.5 and 15 μg/mL OpvE on HaCaT migration assays and found statistically improving capabilities in both assays (Figure 3B,C). Additionally, the transcription of two structural proteins, *COL1A1* and *FN1*, was significantly stimulated. We hypothesize that the level of FN1 in 60 μg/mL OpvE was still lower than that of the assay with 30 μg/mL OpvE (Figure 3D,E) because of the very high growth ratio of the cell. All these experiments provide solid evidence to support that OpvE (<60 μg/mL) could promote skin cell growth and wound healing. Additionally, the computational analysis revealed several key findings, including the PPI network showing the interactions in which those involving ALB and AKT were the most prominent, the GO analysis highlighting significant enrichment in protein phosphorylation, and the KEGG pathway analysis ranking metabolism, cancer, and the PI3K/Akt pathway as the top three enriched pathways (Figure 2). To our knowledge, the high ratio of cell proliferation or migration is dependent on the high metabolite ratio in cells, which can be observed in the pathological process of lethal tumors [28,29]. Numerous studies indicated that the PI3K/Akt pathway is deeply involved in the regulation of cellular functions such as cell migration, cell survival, and other metabolic processes [29,30]. Hence, we demonstrated the regulative capability of OpvE on the PI3K/AKT signaling pathway. The results indicated that in the concentration range of 7.5 to 60 μg/mL, OpvE can stimulate the phosphorylation of PI3K, AKT, and GSK3β (Figure 3F). GSK3β, a key regulator, affects cell differentiation during the fast proliferation of cells [31]. The high level of phosphorylated GSK3β indicated that OpvE treatment can improve cell proliferation and simultaneously keep cell differentiation at normal levels.

During rapid cell proliferation and migration processes, large amounts of free radicals and free reactive oxygen species will be generated [32]. Free radicals, generated by mitochondria, can directly impair physiological and biochemical processes in cells [33]. In our study, we found the free radical scavenging capability of OpvE in a dose-dependent manner. A total of 125 mg/mL OpvE performed a valuable antioxidative activity in DPPH assay tests, by which 97.28% of the DPPH-released free radicals were scavenged (Figure 4A). Furthermore, H_2_O_2_ was applied to induce oxidative stress in the HaCaT cells to verify the antioxidative activity of OpvE (Figure 4B). The pretreatment with OpvE improved the SOD, GSH, and GPX4 levels and inhibited the accumulation of MDA in H_2_O_2-_treated HaCaT cells by regulating the COX2 and NRF2/HO-1/GPX4 pathway (Figure 4C–F). As a key antioxidant axis, the NRF2/HO-1/GPX4 pathway not only protects against skin aging and damage caused by ultraviolet radiation [34] but also plays a broad role in diseases such as Parkinson’s disease [35], periodontitis [36], and non-alcoholic fatty liver disease [37]. It is commonly known that oxidative stress cross-talks with the immuno-inflammation response, which is the key trigger of various pathological processes. Inflammatory stimulation is also a crucial factor tightly associated with skin damage. Skin micro-organisms are the major pathogens that affect skin health, of which the Gram-negative bacterial-released LPS is a popular known inflammation trigger. Our tests proved that OpvE can attenuate the effect of LPS on cell viability and the expression of LPS-stimulated inflammatory factors, such as TNF-α, IL-6, and NF-κB, by regulating TRL4/MYD88 signaling pathways (Figure 5). Also, we were interested in assessing whether OpvE has antibacterial activity. The growth of *S. aureus*, an opportunistic pathogen, could be significantly inhibited by 1.25 mg/mL OpvE. Regrettably, 1.25 mg/mL OpvE also showed obvious cytotoxicity. Hence, we suggested that OpvE is not a valuable antibiotic.

Commercially, the herbal materials for skin-friendly products include *Aloe vera* L. Burm. f. [38], *Centella Asiatica* L. [39], *Calendula officinalis* L. [40], etc. Ferns are an important evolutionary marker of land plants on the earth and occupy an important position in the composition of the flora. There are a lot of ferns used by the Chinese as dietary supplements, even medicines, since ancient times. Even now, *Lycopodium annotinum* L., *Selaginella tamariscina*, *Equisetum hyemale* L., *Lygodium japonicum* (Thunb.) Sw., *Davallia mariesii* Moore ex Bak., *Scutellaria barbata* D. Don, *Pyrrosia lingua* (Thunb.) Farwell, and *Dryopteris crassirhizoma* Nakai (Rhizoma Dryopteris Crassirhizomae) are listed in the Pharmacopoeia of the People’s Republic of China. In comparison to the aforementioned ones, *O. vulgatum* is not a popularly used herb. Based on the findings of our in vitro studies, we confirmed that OpvE (<60 μg/mL) in *O. vulgatum* Linn. has significant skin-friendly activity. However, for clinical application, more evidence is needed. It has been reported to have a high content of unsaturated fatty acids [41], as well as galactoglycerolipids [11], flavonoids [42], and polysaccharides [43]. However, the pharmacochemical characteristics remain unknown. This limits the application of *O. vulgatum*. Our investigation provided some information on the phytochemical and/or pharmacological results of this genus of Pteridophyte. However, our study still has some limitations, as the ingredients of OpvE are still not fully elucidated. Some regulative capabilities of OpvE were not dose-dependent. We should also prepare the proper animal models to evaluate the biological activities of OpvE or the ingredients in OpvE. Nonetheless, we have proved the hair growth-improving capability of OpvE with a mouse model (CN114588184B). However, the specific ingredients and the underlying mechanism are still unclarified.

## 4. Materials and Methods

### 4.1. Chemicals and Reagents

*O. vulgatum* was provided by Ms. Cao Chun, an employee of Novartis Pharmaceuticals Corp, and identified by Prof. DING Zhishan from Zhejiang Chinese Medical University. The collection of plant materials was performed under the relevant guidelines and regulations of the Specification on Good Agriculture and Collection Practices for Medicinal Plants drafted by the China Chamber of Commerce of Medicines & Health Products Importers & Exporters, China. The HaCaT (human immortalized keratinocytes) cell line was obtained from BOSTER (Wuhan, China), and DMSO (dimethyl sulfoxide) and other biochemical reagents were purchased from Huadong Medicinal Technology Co., Ltd. (Hangzhou, China). Fetal bovine serum and DMEM (Dulbecco’s modified eagle medium) high-glucose medium and antibiotics were obtained from Sigma-Aldrich (St. Louis, MO, USA). The CCK-8 (Cell Counting Kit-8) was purchased from Beyotime Biotechnology (Shanghai, China). The ATP (adenosine 5′-triphosphate) assay kit, the DPPH (1,1-diphenyl-2-picryl-hydrazyl radical) assay kit, the CAT (catalase) assay kit, the GSH (glutathione) assay kit, the SOD (superoxide dismutase) assay kit, and the MDA (Malondialdehyde) assay kit were purchased from Nanjing Jiancheng Chemical Industrial Co., Ltd. (Nanjing, China). The RNA preparation kit, the cDNA synthesis kit, the PCR reaction system, and SYBR GREEN were obtained from TaKaRa Bio Inc (Dalian, China). The primers for *COL1A1*, *FN1*, *MYD88*, *TNFα*, *NF-κB*, and *GAPDH* encoding gene polymeric synthesis were supplied by Shanghai Shenggong Biotechnology Co., Ltd. (Shanghai, China). The specific antibodies are listed in Table A4, and the secondary antibodies were procured from BOSTER (Beijing, China). Vancomycin was purchased from Shanghai Yuanye Bio-Technology Co., Ltd. (Shanghai, China). *Staphylococcus aureus* Newman-WT was kindly provided by Prof. Qian Chaodong from Zhejiang Chinese Medical University. The bacterial strain was grown routinely on Luria-Bertani (LB) medium (Oxoid, Hants, UK). Mueller–Hinton (MH) medium (Oxoid, Hants, UK) was used for drug susceptibility testing.

### 4.2. Preparation of O. vulgatum Extract

The forced air oven-dried *O. vulgatum* was pulverized, and 15 g of *O. vulgatum* powder was extracted with 150 mL of slightly boiling 70% ethanol for 1.5 h. Following that, the oleic pigment in crude ethanolic extract was removed by petroleum ether (1:1 *v*/*v*) extraction several times. Then, the OpvE fraction was prepared by ethyl acetate (1:1 *v*/*v*) extraction at least 3 times. Finally, the OpvE fraction was rotary-dried by a rotary steamer (RE2000B, Shanghai Yarong Biochemical Instrument Factory, Shanghai, China) and stored at −80 °C.

### 4.3. UPLC-Q/TOF-MS Analysis

The OpvE powder was dissolved in methanol to create a 1 mg/mL solution. A total of 4 μL 1 mg/mL OpvE methanol solution was loaded into an ACQUITY UPLC BEH C18 column with an ultra-performance liquid chromatography (UPLC) (Waters, Milford, MA, USA) system. The chromatographic conditions are as follows: chromatographic column: Waters ACQUITY UPLC BEH C18 (2.1 mm × 100 mm, 1.7 μm); mobile phase: phase A is 0.1% formic acid acetonitrile and phase B is 0.1% formic acid aqueous solution, using a gradient elution method with a velocity of flow of 0.3 mL/min; tray temperature: 8 °C; column temperature: 40 °C; injection volume: 4 μL; the specific elution conditions are shown in Table A3.

A time-of-flight mass spectrometry (TOF MS) (AB Sciex Pte. Ltd., Framingham, MA, USA) model was performed on mass spectrometry with ion drive turbo v source and ESI positive and negative ion scanning modes. The specific parameters were: ion source gas 1 (Gas1): 55 psi; ion source gas 2 (Gas2): 45 psi; curtain gas (CUR): 35 psi; source temperature: 600 °C; ion spray voltage floating (ISVF): 5500 V in the positive mode while 4500 V in the negative mode; TOF MS scan *m*/*z* range: 100~1500 Da; production scan *m*/*z* range: 25~1500 Da; TOF MS collection: 0.25 s/spectra; product ion scan accumulation time: 0.035 s/spectra. MS/MS data were collected in information-dependent acquisition (IDA) mode with a high sensitivity mode condition. The 60 V delustering potential, 35 eV/−35 eV collision energy, and 15 eV/−15 eV collision energy spread were used in mass detection. The excluded isotopes within 4 Da and the candidate ions to monitor per cycle were 12. The collected molecular mass, as well as the secondary spectrum data, were comparatively screened with SCIEX OS v2.0.4 software, an ingredients database including more than 1500 Chinese herbal medicines. The standard primary accurate mass, isotope distribution ratio, and MS/MS of the compound TCM MS/MS Library in the TCM secondary database were used to tentatively identify the chemical constituents.

### 4.4. Computational Analysis

The Traditional Chinese Medicine Systems Pharmacology database (https://www.tcmsp-e.com/, accessed on 25 July 2023) was used to download the Pubchem Cid ID of the identified chemical compounds in OpvE. Then, we input the PubChem Cid ID into the National Center for Biotechnology Information databases (https://www.ncbi.nlm.nih.gov/, accessed on 25 July 2023) to download the 2D model of the compounds. The specific targets of these compounds were obtained from the Swiss target Prediction databases (http://swisstargetprediction.ch/, accessed on 25 July 2023) and PharmMapper databases (http://www.lilab-ecust.cn/pharmmapper/, accessed on 25 July 2023). Then, these targets were input into Uniprot (https://www.uniprot.org/, accessed on 26 July 2023) to screen the key target genes. The chemical compounds–genes network was structured by inputting target genes into cytoscape 3.10.1. The PPI network was constructed with the STRING database (https://cn.string-db.org/, accessed on 28 July 2023). To visualize the PPI network and represent the key nodes, the cytoscape 3.10.1 software and the cytoNCA algorithm were applied, respectively. Finally, the possible biological activities and capabilities of OpvE were predicted with GO and KEGG pathway analyses by using the DAVID database (https://david.ncifcrf.gov/, accessed on 31 July 2023).

The GO enrichment analysis chart was structured by selecting the smallest top 15 annotations from the biological process (BP), cellular component (CC), and molecular function (MF) categories based on their *p*-values on the bioinformatic platform (http://www.bioinformatics.com.cn/login/, accessed on 8 August 2023). The KEGG enrichment analysis was sorted in descending order of count value, and the top 20 enriched pathways were selected and plotted.

### 4.5. Cell Culture and Treatment

HaCaT cells were cultured in DMEM medium with 10% fetal bovine serum and grown in a 5% CO_2_ incubator (Thermo Fisher Scientific, Waltham, MA, USA) at 37 °C. OpvE (prepared in DMSO, 0~60 µg/mL) was dissolved in DMEM to pretreat HaCaT cells for 16 h. For the oxidative injury protective assay, the OpvE pretreated cells were stimulated with H_2_O_2_ (800 μM) for 12 h. For the inflammatory injury protective assay, the OpvE pretreated cells were incubated in freshly prepared DMEM with LPS (2 μg/mL) for 24 h.

### 4.6. Cell Proliferation Assay

The HaCaT cells growing in freshly prepared DMEM medium contained a gradually increased amount of OpvE, from 0 to 1000 µg/mL for 16–18 h. Six replicates were prepared for each concentration. Each well was measured to show the corresponding proliferation ability of cells under different treatments according to a CCK-8 assay kit (cat. no. C0038).

### 4.7. Wound Healing Assay

The logarithmic growth stage HaCaT cells were plated in 6-well plates. After the well bottom was fully covered, a wound line was scratched with a pipette tip. Each well was washed 3–5 times with phosphate-buffered saline (PBS), and the medium contained 0, 7.5, or 15 μg/L OpvE in each wounding assay. After photographing to record the wound line, the well-plates stayed in the 5% CO_2_ incubator for further cultivation for 24 h. After that, the wound line healing was observed and photographed. Finally, the healing was determined with ImageJ software v1.54f, and the healing ratio was calculated as follows.

Cell scratch healing rate = (N0 − N24)/N0 × 100%

N_0_ indicates the scratch area at time 0, and N_24_ indicates the scratch area at 24 h.

### 4.8. 1,1-Diphenyl-2-Picryl-Hydroxyl Radical (DPPH) Assay

The experimental method referred to the instructions of the DPPH free radical scavenging ability detection kit (cat. no. A153-1-1). A total of 0.4 mL OpvE solutions, with concentrations gradually increased from 15 to 500 µg/mL, was mixed with 0.6 mL of DPPH solution in the dark for 25 min. After centrifuging for 15 min, measured the OD_517_ of the solvents. The DPPH radical scavenging rate was calculated by multiplying the corresponding results in the standard curve and dilution concentration.

### 4.9. Glutathione (GSH), Superoxide Dismutase (SOD), and Malondialdehyde (MDA) Assay

The HaCaTs in various treated assays were harvested and washed with ice-cold PBS. The GSH (cat. no. A006-2-1), SOD (cat. no. A001-3-2), and MDA content (cat. no. A003-4-1) were determined by commercial kits from Nanjing Jiancheng Bioengineering Institute according to the manufacturer’s protocol. Protein content was determined with a bicinchoninic acid (BCA) protein assay kit (Beyotime Biotechnology).

### 4.10. Determination of Antibacterial Activity

The anti-*S. aureus* activity of OpvE was performed by disc diffusion assays. Briefly, 100 μL of *S. aureus* suspension (10^8^ CFU/mL) were seeded on Petri plates containing a Luria-Bertani (LB) agar medium. The paper discs (6 mm in diameter) were separately impregnated with different concentrations (0.6~5 mg/mL) of OpvE in DMSO overnight. After drying, the paper discs were separately placed on the LB agar. Discs containing vancomycin (2 μg/mL) were used as a positive control, and those with DMSO impregnated were used as a negative control. The inoculated plates were incubated for 24 h at 37 °C. Antimicrobial activity was assessed by measuring the diameter of the growth-inhibition zone in millimeters (including a disc diameter of 6 mm).

Anti-*S. aureus* activity assays were performed by the minimum inhibitory concentration (MIC) method and time-kill experiments, as described previously [44]. *S. aureus* was seeded at 5 × 10^5^ cells per well in a 96-well plate containing MH broth with OpvE (0~5 mg/mL). In this experiment, vancomycin was used as a positive control, and sterile MH broth containing 5% DMSO was used as the negative control. After 20 h, the living bacteria in each well were counted by dilution-to-extinction.

### 4.11. Western Blot

Western blotting was conducted as previously [17] described to determine the concentration of protein. Briefly, the HaCaT cells were lysed and centrifuged to obtain cell lysate. The protein concentration was determined, and the cell lysates were loaded and separated with 10–12% SDS-PAGE gel and then transferred onto PVDF membranes. The antibodies applied in this experiment are listed in Table A4. After washing with TBST (Tris-buffered saline + Tween), the membrane was incubated with horseradish peroxidase-conjugated secondary antibodies at room temperature for 1 h. Washing three times with TBST was a necessary step before visualizing with an ECL Plus (Solarbio, Beijing, China, PE0010) kit. The protein level was measured with Image J software.

### 4.12. RT-qPCR

Gene transcription levels were quantified by RT-qPCR. Briefly, total RNA was extracted with the TRIzol reagent (Sangon Biotech, Shanghai, China, B511311) according to the manufacturer’s guidelines. The concentration and integrity of the RNA were identified with a NanoDrop spectrophotometer (Thermo Fisher, Waltham, MA, USA). The mRNA was reverse transcribed with the ReverTra Ace^®^ qPCR RT Master Mix and applied as a template for target gene amplification. A 20 μL real-time PCR assay contained a reverse-transcribed template, amplification mixture, and specific primer pairs (Table A5). With GAPDH as an internal reference, the SYRB green method was employed to measure real-time PCRs using Fast Mix Quantitative RT-PCR Master Mix (Monad, Suzhou, China). Finally, the expression levels of relevant genes were analyzed using the 2^−ΔΔCT^ method.

### 4.13. Statistical Analysis

Data in this research were expressed as means ± standard deviation (SD). The difference between the two groups was statistically analyzed by unpaired *t*-test using GraphPad Prism 8.3.0 software (GraphPad Software Inc., La Jolla, CA, USA). A *p*-value < 0.05 was considered significantly different.

## 5. Conclusions

In summary, our research identified the chemical composition of the ethyl acetate fraction of *O. vulgatum* and proved the beneficial impact of low doses (7.5~15 μg/mL) of OpvE for epidermal cell proliferation and migration-promoting activities by regulating the PI3K/AKT signaling pathway. OpvE (7.5~60 μg/mL) has antioxidative and anti-inflammatory activities, primarily through the regulation of the Nrf2/HO-1/COX2 signaling axis and the TLR4/MYD88 signaling pathway, respectively. While a high dose of OpvE maintained the anti-*S. aureus* efficacy and free radical scavenging capacity, it should be noted that these elevated doses concurrently exhibited cytotoxic effects. This work highlighted the phytochemical characteristics and some bioactivities, as well as the underlying mechanism, which would support further studies and application of *O. vulgatum*.

## 6. Patents

Tang, L.; Yan, F.; Bin, D. The extract, preparation method and application of *Ophioglossum vulgatum* Linn. CN114588184B, 2023-08-25 [14].

## Figures and Tables

**Table 1:** Anti-*S. aureus* activity of OpvE determined by the disc diffusion.

Inhibition Zone Diameters (mm)				
OpvE/Concentrations (mg/mL)			Vancomycin (μg/mL)	
5.00	2.50	1.25	0.60	2.00
12.6 ± 0.97	9.3 ± 0.43	8.3 ± 0.16	7.625 ± 0.33	17.475 ± 0.36

## Data Availability

Data are contained within the article.

## Abbreviations

The following abbreviations are used in this manuscript:

Akt	Protein kinase B
ATP	Adenosine triphosphate
BP	Biological function
CC	Cell component
CCK-8	Cell counting kit-8
COL1A1	Collagen type I alpha 1 Chain
COX2	Cyclooxygenase-2
Ctrl	Control
DPPH	1,1-diphenyl-2-picryl-hydrazyl radical
FN1	Fibronectin 1
GO	Gene Ontology
GSH	Glutathione
GSK3-β	Glycogen synthase kinase 3 beta
H_2_O_2_	Hydrogen peroxide
HaCaT	Human keratinocyte
HO-1	Heme oxygenase 1
IL-6	Interleukin-6
KEGG	Kyoto encyclopedia of genes and genomes
LPS	Lipopolysaccharides
MDA	Malondialdehyde
MF	Molecular function
MIC	Minimum inhibitory concentration
MYD88	Myeloid differentiation primary response protein
NF-κB	Nuclear factor-kappa-B
Nrf2	Nuclear factor erythroid 2-related factor 2
OpvE	Ethyl acetate fraction of *O. vulgatum*
p-AKT	Phospho-protein kinase B
PCR	Polymerase chain reaction
p-GSK3-β	Phospho-glycogen synthase kinase 3 beta
PI3K	Phosphatidylinositol 3-kinase
p-NF-κB	Phospho-nuclear factor-kappa-B
p-PI3K	Phospho-phosphatidylinositol 3-kinase
SOD	Superoxide dismutase
TIC	Total ion chromatograms
TLR4	Toll-like receptor 4
TNF-α	Tumor necrosis factor-alpha
UPLC-Q/TOF-MS	Ultra-high performance liquid chromatography-quadrupole time-of-flight mass spectrometry
UVB	Ultraviolet radiation B

## Appendix A

**Table A1:** The identified ingredients in positive ion mode.

No.	Component Name	Area	Retention Time	Formula	Precursor Mass	Found at Mass	Mass Error (ppm)	Library Score	Isotope Ratio Difference
1	Protocatechuic acid	182,600	1.39	C_7_H_6_O_4_	153.019	153.0194	0.2	94.7	1.2
2	Ligustroflavone	89,630	1.65	C_33_H_40_O_18_	723.214	723.2141	−0.2	71.9	1.2
3	Rutin	21,570	1.68	C_27_H_30_O_16_	609.146	609.1457	−0.6	97.6	2.1
4	Aempferol-3-O-rutinoside	53,690	1.96	C_27_H_30_O_15_	593.151	593.1511	−0.1	88.4	2.9
5	p-Coumaric acid	46,360	2.01	C_9_H_8_O_3_	163.04	163.04	−0.4	91.8	1.2
6	Quercitrin	116,500	2.01	C_21_H_20_O_11_	447.093	447.0932	−0.2	93.2	2.9
7	Afzelin	43,890	2.4	C_21_H_20_O_10_	431.098	431.0982	−0.3	87.4	1.8
8	Tiliroside	26,170	3.04	C_30_H_26_O_13_	593.13	593.13	−0.1	80.7	3.3
9	Luteolin	699,800	3.3	C_15_H_10_O_6_	285.04	285.0407	0.9	95.1	0.7
10	Tectoridin	25,430	3.55	C_22_H_22_O_11_	461.109	461.1088	−0.4	90.2	3.1
11	Isorhamnetin	9 × 10^6^	3.7	C_16_H_12_O_7_	315.051	315.0513	0.8	76.7	0.3
12	Apigenin	72,740	4.14	C_15_H_10_O_5_	269.046	269.0457	0.5	95.7	0.6
13	Pedunculoside	15,070	4.18	C_36_H_58_O_10_	695.401	695.4015	0.4	94.6	3.7
14	Physcion	6703	4.38	C_16_H_12_O_5_	283.061	283.0611	−0.4	96.6	3.8
15	Pectolinarigenin	28,180	6.88	C_17_H_14_O_6_	313.072	313.0717	−0.3	88.8	0.6
16	Liriope muscari baily saponins C	11,070	7.02	C_44_H_70_O_17_	915.46	915.4592	−0.3	88.5	4.8
17	Asiatic acid	220,500	7.73	C_30_H_48_O_5_	487.343	487.3428	−0.2	86.5	1.6

**Table A2:** The identified ingredients in negative ion mode.

No.	Component Name	Area	Retention Time	Formula	Precursor Mass	Found at Mass	Mass Error (ppm)	Library Score	Isotope Ratio Difference
1	Betaine	561,100	1.04	C_5_H_11_NO_2_	118.086	118.0864	1.3	95.8	0.7
2	Luteoloside	32,670	1.7	C_21_H_20_O_11_	449.108	449.109	2.6	95.7	3.4
3	Luteolin	382,700	3.29	C_15_H_10_O_6_	287.055	287.0555	1.8	100	0.1
4	Liquiritin	33,360	3.79	C_21_H_22_O_9_	419.134	419.1346	2.2	73.7	3.1
5	6-Gingerol	191,700	12	C_17_H_26_O_4_	295.19	295.1909	1.6	73	1.8

**Table A3:** Chromatographic elution conditions by UPLC-Q-TOF/MS.

Time (min)	Flow (mL/min)	A% (0.1% Formic Acid Acetonitrile)	B% (0.1% Formic Acid Aqueous Solution)
0	0.3	25	75
7.5	0.3	60	40
15	0.3	80	20
25	0.3	99	1
28	0.3	99	1

**Table A4:** List of antibodies for WB.

Antigen	Dilution	Vendor	Catalog No.
p-PI3K	1:1000	Cell Signaling Technology (Danvers, MA, USA)	17366
PI3K	1:1000	Cell Signaling Technology	4292
p-AKT	1:1000	BOSTER (Pleasanton, CA, USA)	BM4721
AKT	1:1000	BOSTER	A00024-2
p-GSK3β	1:1000	BOSTER	BM4837
GSK3β	1:10,000	BOSTER	A00791-3
TLR4	1:1000	BOSTER	AF8208
NF-κB	1:1000	BOSTER	AF0246
p-NF-κB	1:1000	Cell Signaling Technology	3033
TNF-α	1:1000	BOSTER	BA0131
IL-6	1:1000	BOSTER	BA0562-2
NRF2	1:1000	BOSTER	A00078-1
COX2	1:1000	BOSTER	A00084
HO-1	1:1000	BOSTER	PB0050
GPX4	1:1000	BOSTER	A02059-1
β-actin	1:5000	BOSTER	BM3873
GAPDH	1:5000	BOSTER	BM3876

**Table A5:** List of primers for real-time PCR.

Gene Name	Forward Primer	Reverse Primer
*COL1A1*-H	TAGGGTCTAGACATGTTCAGCTTTG	CGTTCTGTACGCAGGTGATTG
*FN1*-H	AGGAAGCCGAGGTTTTAACTG	AGGACGCTCATAAGTGTCACC
*MYD88*-H	GGCTGCTCTCAACATGCGA	CTGTGTCCGCACGTTCAAGA
*TNFα*-H	CCTCTCTCTAATCAGCCCTCTG	GAGGACCTGGGAGTAGATGAG
*NF-κB*-H	AACAGAGAGGATTTCGTTTCCG	TTTGACCTGAGGGTAAGACTTCT
*GAPDH*-H	GCACCGTCAAGGCTGAGAAC	TGGTGAAGACGCCAGTGGA
